# Prostate examination among adult and elderly subjects in southern Brazil: a cross-sectional population-based study

**DOI:** 10.1590/1516-3180.2020.0197.R1.03092020

**Published:** 2020-11-02

**Authors:** Kevin Francisco Durigon Meneghini, Hsu Yuan Ting, Samuel Carvalho Dumith

**Affiliations:** I Medical Student, School of Medicine, Fundação Universidade Federal do Rio Grande (FURG), Rio Grande (RS), Brazil.; II MD, MSc, PhD. Assistant Professor, Department of Surgery, School of Medicine, Fundação Universidade Federal do Rio Grande (FURG), Rio Grande (RS), Brazil.; III BSc, MSc, PhD. Assistant Professor, Postgraduate Program on Health Sciences, Fundação Universidade Federal do Rio Grande (FURG), Rio Grande (RS), Brazil.

**Keywords:** Prostate, Epidemiology, Prostate-specific antigen, Digital rectal examination, Men's health, Prostate cancer, Screening, Southern Brazil

## Abstract

**BACKGROUND:**

Population-wide screening for prostate cancer remains a controversial topic, given the need for an individualized approach to patients regarding the risks and benefits of prostate-specific antigen testing and digital rectal examination.

**OBJECTIVE:**

The aim of this study was to investigate the prevalence of, and factors associated with, prostate examination among men aged 45 or older.

**DESIGN AND SETTING:**

Cross-sectional population-based study developed in the city of Rio Grande (RS), Brazil.

**METHODS:**

The outcome of interest was a history of prostate examination (prostate-specific antigen testing or digital rectal examination). The following independent variables were analyzed: age group, skin color, marital status, schooling, economic level, leisure-time physical activity, smoking habits, excessive alcohol consumption, overweight, health insurance, visits to the doctor during the preceding year, hypertension and diabetes. After a two-stage sampling process, the final sample consisted of 281 male individuals.

**RESULTS:**

The prevalence of a history of prostate-specific antigen testing or digital rectal examination was 68.3% (95% confidence interval (CI): 62.2 to 74.5). The highest prevalence rates were observed among men aged 70 years or older (88%) and the lowest among smokers (36%). The following characteristics were found to be associated with the outcome: advanced age; marital status other than single; more schooling and higher economic status; practicing physical activity; non-smoking habits; overweight; having health insurance; and having visited a doctor during the preceding year.

**CONCLUSION:**

Approximately two thirds of the study population had been screened for prostate examination, mostly older individuals, with higher socioeconomic status and a healthier lifestyle.

## INTRODUCTION

Population-wide screening for prostate cancer remains a controversial topic, given the need for an individualized approach to patients regarding the risks and benefits of prostate-specific antigen testing and digital rectal examination. Treatment of prostate cancer may prove challenging because of matters such as biopsy procedures, which may lead to local complications (e.g. infection); and also because of the possibility of sexual impotence and urinary incontinence secondary to treatment.^1^^,^^2^

The combination of prostate-specific antigen testing and digital rectal examination has been considered to be an effective approach, since 18% to 45% of tumors would not have been diagnosed, had one of these two methods not been performed.^3^ The American Cancer Society advises that, among men whose life expectancy exceeds 10 years, screening should be done annually, through informed consent. This should be started at the age of 50 years for those at moderate risk; at the age of 45 for those at high risk (afro-descendants and individuals with a history of prostate cancer in first-degree family members at ages younger than 65 years); and at the age of 40 for those at very high risk (multiple family members diagnosed with prostate cancer before the age of 65).^4^

In an official note, in 2017, the Brazilian Society of Urology advised that from the age of 50 years onwards, the male population should seek a specialist annually, for assessment and discussion of the risks and benefits of prostate cancer screening. The Brazilian Society of Urology recommends that men aged 45 who present risk factors should undergo screening for prostate cancer; but for individuals aged 75 and older, this is valid only for those with life expectancy greater than 10 years.^2^

In guidelines issued in 2013, the American Urological Association was in favor of screening for prostate cancer among individuals aged 55 to 69 years, if they so desired, and suggested that a two-year interval between examinations would preserve the benefits and reduce overdiagnosis and false positives.^5^ In 2018, the United States Preventive Services Task Force also indicate screening for the age group from 55 to 69, based on an analysis of risks versus benefits.^6^

However, like the Australian Federal Department of Health and the National Screening Committee in the United Kingdom, the Brazilian Ministry of Health does not recommend routine screening and advises that individuals in the male population who are spontaneously willing to get tested should be widely informed about the associated risks and benefits.^7^^,^^8^

In this study, we determined the profile and sociodemographic context of individuals undergoing screening for prostate cancer, along with their level of awareness regarding prostate health.

## OBJECTIVES

The aim of this study was to investigate the prevalence of, and factors associated with, prostate examination among men aged 45 years or older in the city of Rio Grande (RS), Brazil.

## METHODS

This was a cross-sectional population-based study, which formed part of a larger project named “Health of the population of Rio Grande”. The questionnaire from this project was applied by nine trained interviewers, who were supervised by ten postgraduate students. This interview process was coordinated by two professors of postgraduate programs at the Fundação Universidade Federal do Rio Grande (FURG). The criteria for interviewer selection were the following: female sex; at least high-school education completed; available in the evenings and on the weekends; attendance at training; and approval in tests during the training. It was decided to select only female interviewers because the potential subjects were more likely to receive them and feel safer to open their houses for them. During the data collection, four interviewers continued to work until the end of the data collection and conducted about 80% out of all the interviews.

Demographic census data indicated that the target population for the study comprised 138,996 individuals aged at least 18 years.^9^ The parameters used for the prevalence outcome calculation were the following: an estimated prevalence of 10% with a range of error of two percentage points and a 95% confidence interval, thus totaling 860 individuals. To this, 50% was added to account for the design effect. In relation to associated factors, the calculation was as follows: an estimated prevalence outcome of 10% with a 95% confidence interval and power of 80%. Furthermore, a prevalence ratio of 2,0 and exposure frequency range from 20% to 60% were used, thus totaling 784 subjects. To this, 50% was added to account for the design effect, which was considered to be 1.5; and, to this, another 15% was added with the aim of minimizing confounding factors. In this manner, a total sample size of 1,294 individuals was reached. To this, another 10% was added to account for possible missing of interviews or refusal to participate. Hence, the final sample size became 1,423 eligible subjects.

The sampling process was carried out in two stages, considering firstly census tracts and secondly households and individuals. Seventy-two out of the 293 eligible census tracts (25%) were systematically selected, and an average of 10 households per tract was then selected. An average of two individuals aged at least 18 years was estimated per household. Hence, the total number of 1,423 individuals corresponded to an estimate of 710 households. To minimize the design effect, more census tracts and fewer households were preferred. Further methodological details can be found elsewhere.^9^

Out of the 1,423 individuals who were found to be eligible to be included in the survey “Health of the population of Rio Grande” after the sampling process, 1,300 were interviewed. Thus, the sample loss was around 10%.

In the present study, the data analysis was restricted to eligible male individuals aged 45 years or older, living in the urban area of Rio Grande (n = 281). Those among the 1,423 individuals in the original sample who were institutionalized in nursing homes, hospitals or prisons, or who were physically and/or cognitively unable to answer the questionnaire, were excluded from the analysis.^9^

The main dependent variable was a self-reported history of prostate examination at least once in a lifetime. The secondary outcome was a history of prostate-specific antigen testing and digital rectal examination. The following independent variables were analyzed: age group, skin color, marital status, schooling, economic status, leisure-time physical activity, smoking habits, excessive alcohol consumption, overweight, health insurance, visits to the doctor during the preceding year, hypertension and diabetes. The study participants' economic status was assessed through an asset index that was determined by means of analysis on the main components of specific household goods. This index took into consideration the participants' possession of specific household goods and their household characteristics. Data on leisure-time physical activity were collected through the International Physical Activity Questionnaire and were dichotomized into “yes” or “no”.^10^ Excessive alcohol consumption was defined as ingestion of five or more standard drinks for men and four or more standard drinks for women on a single occasion.^11^ Excess weight was defined as having a body mass index above 24.9 kg/m², based on self-reported weight and height data. Information on hypertension and diabetes was collected based on a self-reported medical diagnosis.

For data quality control, some key questions from the questionnaire were applied again to 10.5% of the sample, in order to verify whether the interviews were really conducted. From this process, an average kappa index value of 0.80 was obtained. The questionnaires were then coded, reviewed and entered twice into the Epi-Data 3.1 software (EpiData Association, Odense, Denmark). Subsequently, the data were transferred to the Stata 11.2 statistical software package (Stata Press, College Station, Texas, United States) for exploratory analysis, transformation and categorization of variables. A univariate analysis was performed using absolute and relative frequencies. Bivariate and multivariate analyses were performed using Poisson regression, to take the effect of the sample design into consideration. The significance level was taken to be 5% in all two-tailed statistical tests.

This research project had previously been approved by the local public university research ethics committee, under the number 20/2016, dated March 18, 2016. The study volunteers signed an informed consent form to authorize their participation, or consented to this by fingerprinting the form after it had been read aloud to them.

## RESULTS

The final sample size consisted of 281 male individuals aged 45 years or older (mean: 59.3 years; standard deviation (SD): 10.6; maximum age: 96 years). The sample design effect for the prostate examination variable was 1.23 (intraclass correlation coefficient = 0.02).

Most of these individuals were white (86%); were married, divorced or widowed (76%); had 0 to 8 years of schooling (56%); and were not practicing leisure-time physical activity (65%). One fifth (21%) of them were smokers; 14% had consumed alcohol in excess within the previous 30 days; 62% were overweight; 52% had health insurance; three quarters (75%) had visited a doctor during the preceding year; and 39% reported a medical diagnosis of hypertension and 12%, diabetes (Table 1).

**Table 1:** Description of the sample of male individuals aged 45 years or older, living in the urban area of Rio Grande (RS), who either had or had not undergone prostate examinations, surveyed in 2016

Variable		n	Had undergone prostate examination (%)	Had not undergone prostate examination (%)
**Age groups (years)**				
	45-49	60	51.7	48.3
	50-59	98	66.3	33.7
	60-69	72	70.8	29.2
	≥ 70	51	88.2	11.8
**Skin color**				
	White	241	69.3	30.7
	Others	40	62.5	37.5
**Marital status**				
	Single	68	47.1	52.9
	Married, widowed, separated or divorced	213	75.1	24.9
**Schooling (years)**				
	0-8	158	62.0	38.0
	9-11	65	73.3	26.7
	≥ 12	57	82.5	17.5
**Economic status (in terciles)**				
	Poorest	94	54.3	45.7
	Intermediate	82	73.3	26.7
	Richest	105	82.5	17.5
**Leisure-time physical activity**				
	No	183	59.6	40.4
	Yes	97	85.6	14.4
**Smoking habit**				
	No	223	76.7	23.3
	Yes	58	32.6	67.4
**Excessive alcohol consumption**				
	No	242	69.0	31.0
	Yes	38	63.2	36.8
**Overweight**				
	No	105	55.2	44.8
	Yes	173	75.7	24.3
**Health insurance**				
	No	134	52.2	47.8
	Yes	147	83.0	17.0
**Visit to a doctor during the preceding year**				
	No	71	43.7	56.3
	Yes	210	76.7	23.3
**Hypertension**				
	No	172	62.2	37.8
	Yes	109	78.0	22.0
**Diabetes**				
	No	248	66.5	33.5
	Yes	33	81.8	18.2
	Total	281		

The prevalence of men who had undergone prostate-specific antigen testing or digital rectal examination in their lifetimes was 68.3% (95% confidence interval (CI): 62.2 to 74.5). Of these, 45.3% (n = 87) had been tested through both methods (prostate-specific antigen testing and digital rectal examination). The highest prevalence rates of for prostate examination were observed among men aged 70 years or older (88.2%) and the lowest among smokers (36.2%) (Table 2). The following characteristics were found to be associated with the outcome: advanced age; being married, divorce, or widowed; having 12 or more years of schooling; having higher economic status; practicing leisure-time physical activity; non-smoking habits; overweight; having health insurance; having visited a doctor during the preceding year; and having a diagnosis of hypertension and/or diabetes (Table 2). However, through the adjusted analysis, the association between the outcome and hypertension and diabetes ceased to be statistically significant.

**Table 2:** Prevalence of prostate examination among male individuals aged 45 years or older who were living in the urban area of Rio Grande (RS), surveyed in 2016 (n = 281)

Variable		Crude analysis PR (95% CI)	Adjusted analysis PR (95% CI)
**Age groups (years)**			
	45-49	1.00	1.00
	50-59	1.28 (0.99-1.67)	1.24 (0.98-2.59)
	60-69	**1.37 (1.02-1.85)** §	**1.35 (1.02-1.78)** §
	≥ 70	**1.71 (1.28-2.27)** §	**1.68 (1.29-2.19)** §
**Skin color**			
	White	1.11 (0.88-1.40)	1.07 (0.85-1.36)
	Others	1.00	1.00
**Marital status**			
	Single	1.00	1.00
	Married, widowed, separated or divorced	**1.60 (1.26-2.02)** §	**1.38 (1.09-1.74)** §
**Schooling (years)**			
	0-8	1.00	1.00
	9-11	1.17 (0.96-1.41)	1.06 (0.88-1.28)
	≥ 12	**1.33 (1.13-1.57)** §	**1.24 (1.02-1.51)** §
**Economic status (in terciles)**			
	Poorest	1.00	1.00
	Intermediate	1.21 (0.94-1.58)	1.19 (0.91-1.57)
	Richest	**1.53 (1.23-1.89)** §	**1.36 (1.07-1.74)** §
**Leisure-time physical activity**			
	No	1.00	1.00
	Yes	**1.44 (1.25-1.65)** §	**1.22 (1.08-1.37)** §
**Smoking habits**			
	No	**2.11 (1.54-2.91)** §	**1.58 (1.18-2.12)** §
	Yes	1.00	1.00
**Excessive alcohol consumption**			
	No	1.09 (0.86-1.40)	0.92 (0.72-1.17)
	Yes	1.00	1.00
**Overweight**			
	No	1.00	1.00
	Yes	**1.37 (1.15-1.64)** §	**1.31 (1.10-1.55)** §
**Health insurance**			
	No	1.00	1.00
	Yes	**1.59 (1.33-1.89)** §	**1.35 (1.14-1.60)** §
**Visit to a doctor during the preceding year**			
	No	1.00	1.00
	Yes	**1.76 (1.35-2.29)** §	**1.44 (1.15-1.80)** §
**Hypertension**			
	No	1.00	1.00
	Yes	**1.25 (1.07-1.46)** §	0.98 (0.86-1.12)
**Diabetes**			
	No	1.00	1.00
	Yes	**1.23 (1.01-1.50)** §	1.04 (0.87-1.24)

§ Statistically significant (P < 0.05); PR = prevalence ratio; CI = confidence interval.

Among the 68% who had been screened (n = 192), our findings showed that older individuals with higher economic status were more likely to have been tested using both methods (versus only using one of them). Analysis on the likelihood of having been tested using both methods (prostate-specific antigen testing and digital rectal examination) versus not having been tested, the associated factors were the same as those for having been tested using one of these two methods (data not shown).

## DISCUSSION

This study reports the factors associated with prostate-specific antigen testing and digital rectal examination among men aged 45 years and older. Our findings indicated that seven in every ten individuals reported a history of having undergone prostate examination in their lifetimes. After adjustment for possible confounders, the following characteristics remained associated with the outcome: advanced age; marital status other than single; more schooling; being in the upper tercile of economic status; practicing physical activity; non-smoking habits; overweight; having health insurance; and having visited a doctor during the preceding year.

The prevalence rate of prostate-specific antigen testing observed in our study (33.1%) was similar to, or greater than, the rates that have been reported in developed countries. A study carried out in Milan, Italy, between 1999 and 2000, revealed that over 300,000 men had been tested for prostate-specific antigen, which corresponded to a prevalence rate of 26.9%. When only individuals younger than 50 years were considered, the prevalence rate of prostate-specific antigen testing increased to 34%, which the authors of that study considered to be high coverage of the population.^12^

An analysis on data gathered through the Behavioral Risk Factor Surveillance System (BRFSS) in the United States in 2012 and 2014, on a population of 158,103 men aged 40 to 64 years who had been tested for prostate-specific antigen in the previous year, indicated that the prevalence rates of prostate examinations in 2011 and 2013 were 24.4% and 22.3%, respectively.^13^ In addition, a study on data from the Dominican Republic Demographic and Health Survey (DRDHS, 2013), on a population of 3,272 men aged 40 to 60 years old, found that 30.6% of them had been screened preventively for prostate cancer (prostate-specific antigen testing or digital rectal examination) at some point in their lifetimes.^14^ That prevalence rate was less than half of the rate found in our study (68.3%).

In Brazil, three cross-sectional studies were carried out between 2001 and 2007 to determine the coverage of prostate examinations (prostate-specific antigen testing or digital rectal examination) in the city of São Paulo, the coastal region around Santos (Baixada Santista) and the remainder of the state of São Paulo. The studies had heterogeneous designs: two of them were population-based surveys and the third used a research instrument that had been designed specifically for that study. The findings from these studies were as follows:

- In the city of São Paulo, the prevalence rate of prostate examination in the city of São Paulo was 47%, based on a sample of 540 men older than 18 years. Although significant, that prevalence was lower than what we observed in our study. Furthermore, a high proportion of non-coverage among individuals under 50 years of age was expected, as shown in our results, which was close to one in every two individuals.^15^
- The Multicenter Health Survey of the State of São Paulo (Inquérito de Saúde no Estado de São Paulo, ISA-SP) indicated that 55.6% out of 992 men aged at least 50 years had been screened for prostate cancer. Of these, 73% had undergone prostate-specific antigen testing, 62% digital rectal examination and 22% both examinations. Among all the examinations, 50% had been performed in the previous year, probably due to the predominance of individuals aged over 60 in the sample.^3^
- In the Baixada Santista, a study conducted among 927 respondents aged 40 years or older showed that 56.5% of them had been tested for prostate-specific antigen at least once in their lifetimes.^16^

The risk factors for development of prostate cancer include the following:

1. Age – in Brazil, out of every ten diagnoses, nine are among men older than 55 years, particularly those older than 65 years (85%). In contrast, the American Cancer Society has estimates that six out of every ten diagnoses occur in men aged at least 65 years.^1^^,^^14^^,^^17^
2. Ethnicity – Afro-descendants.^14^^,^^17^
3. Family history of prostate cancer – defined as a father or sibling diagnosed before the age of 60.^1^
4. Overweight and obesity.^1^

On the other hand, the main protective factors against prostate cancer are the following: healthy eating, physical activity practice, adequate body weight, non-smoking habits and no alcohol consumption.^1^

In the present study, the group of men aged at least 70 years had been more frequently screened for prostate cancer through prostate-specific antigen testing or digital rectal examination. Importantly, one in every two men had been tested by means of both prostate-specific antigen testing and digital rectal examination. These findings are in line with the tendency shown in the ISA-SP survey, which reported that the prevalence was around 70% for this age group.^18^ Due to comorbidities resulting from aging, individuals aged 70 and older are more frequently in contact with healthcare services and, therefore, are more likely to undergo preventive examinations. In addition, aging has also been associated with benign prostatic hyperplasia, which gives rise to a need for prostate-specific antigen testing and digital rectal examination.^18^

The Dominican Republic Demographic and Health Survey (DRDHS, 2013) indicated a trend towards a higher frequency of prostate examinations with aging,^14^ but only from the age of 60 years onwards. Conversely, Americans aged 50 to 59 years were screened approximately 2.5 times more frequently for prostate cancer than were older individuals.^19^

In our study, there was a significant association between marital status other than single (married, divorced or widowed) and higher prevalence of preventive screening for prostate cancer. This was in line with the findings from a study conducted in the Caribbean region.^14^ We further observed that this did not occur only in relation to prostate-specific antigen testing, unlike what was reported in studies carried out in São Paulo and in the United States.^13^^,^^16^ These last two studies also showed a positive association with prostate examination among individuals who had a steady partner or a casual partner, or who were widowed or divorced.^13^^,^^16^ In the same way, in our study population, the lowest prevalence rates for the outcome were observed among single individuals. Conversely, in another study, it was reported that Americans who had never married or were single underwent more preventive examinations for prostate cancer.^19^

Factors such as more schooling, higher income, having health insurance and having visited a doctor during the preceding year are well established in the literature as predictive of undergoing prostate examination.^13^^,^^14^^,^^16^^,^^18^^,^^19^ In our study, more schooling and higher income were positively associated with undergoing screening for prostate cancer, while having not visited a doctor during the preceding year proved to be an important negative factor for prostate examination (prostate-specific antigen testing and digital rectal examination), as expected.

Consistent with the findings from the ISA-SP survey, non-smoking men had been screened for prostate cancer more often,^18^ while lower prevalence rates were observed among smokers. In our study, overweight was also significantly associated with the outcome. We reasoned that the higher prevalence of prostate examinations among overweight or obese men was because they sought healthcare on a frequent basis through awareness that their condition was a risk factor for prostate cancer.^1^ In contrasting studies, one carried out in the United States demonstrated that not being overweight was a factor associated with being screened for prostate cancer, while another conducted nationwide in Brazil showed that this characteristic was not statistically significant.^18^^,^^19^

Our study has important limitations that need to be considered, namely:

1. It was impossible to establish a causal relationship due to the cross-sectional study design, and because of biases of memory and information regarding self-reported data. However, it is important to note that such an approach has been considered effective for population-wide surveys, to monitor cancer-related knowledge and preventive practices.^20^
2. Because of the scope of the base study (“Health of the population of Rio Grande”), it was not possible to provide any details concerning the clinical outcomes that led to use of prostate-specific antigen testing and digital rectal examination, or to scrutinize the results further.
3. This study only reflected the situation of a small area in the state of Rio Grande do Sul. Therefore, the capacity to generalize these results to the metropolitan regions of Brazil or to the entire country is limited.

To our knowledge, this is one of the few population-wide studies in the literature to have investigated the prevalence of, and factors associated with, prostate cancer screening. To date, there are no international guidelines in this field, in contrast to the situation regarding mammography and cervical screening among women. Hence, the advisory level for the recommendation that the male population should undergo preventive prostate screening is only at Grade C level, i.e. that this should be discussed individually.^5^

## CONCLUSION

Approximately two thirds of the study population had been screened for prostate cancer. These individuals were mostly older, with higher socioeconomic status, healthier lifestyle and frequent use of healthcare services.
